# Health-related quality of life in children with congenital adrenal hyperplasia

**DOI:** 10.1186/s12955-017-0769-7

**Published:** 2017-10-06

**Authors:** Alyssa Halper, Mary C. Hooke, Maria Teresa Gonzalez-Bolanos, Nancy Vanderburg, Thang N. Tran, Jane Torkelson, Kyriakie Sarafoglou

**Affiliations:** 1 University of Minnesota Masonic Children’s Hospital, 2450 Riverside Ave. East Bldg, Rm MB671, Minneapolis, MN 55454 USA; 2 University of Minnesota School of Nursing, Minneapolis, MN USA; 3 University of Minnesota College of Pharmacy, Minneapolis, MN USA; 4 Minnesota Department of Health, St. Paul, MN USA

**Keywords:** Congenital adrenal hyperplasia, Quality of life, Pediatrics, Glucocorticoids, Hydrocortisone, Fatigue, Cortisol, Androgen

## Abstract

**Background:**

Children with congenital adrenal hyperplasia (CAH) require life-long glucocorticoid replacement and have daily intermittent hyper/hypocortisolemia and hyperandrogenemia. Health-related quality of life (HRQL) is important for understanding the impact the disease and therapy have on physical, mental, emotional, and social functioning. Little is known about HRQL in CAH. We compared HRQL in children with CAH to healthy norms and examined how these scores related to physiologic variables.

**Methods:**

A cross-sectional study examined 45 patients (mean age 8.2(4.5) years). Thirty-two self-reported their quality of life (QoL) on the PedsQL™ Generic Core Scale and PedsQL™ Fatigue Scale, and 44 parents completed a parent report. Bone age Z-scores were calculated from the most recent bone age.

**Results:**

Children with CAH did not report lower QoL than healthy norms. However, their parents reported lower overall QoL and fatigue scores than parents of healthy norms. Children with CAH rated sleep poorer than their parents. QoL scores did not differ by sex or CAH subtype and were not associated with total daily hydrocortisone dose. Bone age Z-scores were negatively associated with child-reported emotional health and cognitive fatigue.

**Conclusions:**

Parents of children with CAH reported a negative impact of disease on their children’s QoL, but their children did not. The negative associations between bone age Z-scores and emotional health and cognitive fatigue suggest an impact from chronic hypocortisolemia and hyperandrogenemia.

## Background

Congenital adrenal hyperplasia (CAH) due to 21α-hydroxylase deficiency is a form of adrenal insufficiency characterized by impaired cortisol synthesis and excessive adrenal androgen production. Depending on the severity, CAH is classified as either classic (severe phenotype) or non-classic (mild phenotype). The classic phenotype is subdivided into simple-virilizing and salt-wasting based on whether there is an adequate or deficient aldosterone production. Treatment for CAH involves life-long glucocorticoid replacement. In growing children, hydrocortisone is the recommended glucocorticoid at 10-15 mg/m^2^/day [1]. Due to the short half-life of hydrocortisone and variable inter-individual glucocorticoid receptor sensitivity, children with CAH are exposed to daily non-physiologic, non-circadian cortisol profiles with intermittent hyper/hypocortisolemia and hyperandrogenemia throughout each day [2]. Fluctuating cortisol and adrenal androgen concentrations may impact quality of life (QoL) and may affect energy levels in children with CAH. There have only been two studies that have examined overall QoL in children with CAH [3, 4], and none have examined fatigue. In addition, no studies have correlated fatigue and overall QoL with hydrocortisone dosing or measures of androgen exposure and disease control in children, such as bone age Z-scores.

The aims of this study were to compare health-related quality of life (HRQL) in children with CAH treated with hydrocortisone and their parents with healthy norms, and to examine the relationship between these scores and physiologic variables. We report scores from both the validated PedsQL™ Generic Core Scale and the PedsQL™ Fatigue Scale.

## Methods

### Participants

Children with CAH and their parents completed the PedsQL™ measurements during their routine clinic visit at the University of Minnesota Masonic Children’s Hospital Multidisciplinary CAH Clinic before consultation and without the investigator present. Participants included 45 children (25 females, 20 males) diagnosed with CAH (25 salt-wasting, 13 simple-virilizing, and 7 non-classic) with a mean age of 8.22 years (2-19 years) [Table 1]. Diagnosis of CAH was confirmed by biochemical and molecular testing. Children between 5 and 19 years of age (*n* = 32) completed the self-report measures, and parents of children between 2 and 19 years of age (*n* = 44) rated their child’s HRQL on a parent proxy-report form. The University of Minnesota Institutional Review Board approved the study, and informed consents from all participants and assents for children older than 7 years were obtained.

**Table 1:** Sociodemographic and medical profiles of Children with CAH

Variables	N (%)	Mean (SD)
Sex		
Male	20 (44)	
Female	25 (45)	
Race		
Caucasian	35 (76)	
Hispanic Latino	3 (7)	
Mixed Race	5 (11)	
Other	2 (4)	
CAH Classifications		
Salt-wasting	25 (56)	
Simple-virilizing	13 (29)	
Non-classic	7 (15)	
Age at time of study (years)		8.22 (4.53)
Hydrocortisone Dose		
Total Dose (mg/m^2^/day)		10.26 (2.79)
Morning Dose (mg/m^2^/day)		6.01 (2.18)
Bone Age Z-scores		2.01 (2.79)
Height Z-scores		0.62 (1.36)

*CAH* congenital adrenal hyperplasia

### Methods

QoL was measured through the validated PedsQL™ Generic Core Scale and the PedsQL™ Fatigue Scale [5–8].

The PedsQL™ Multidimensional Generic Core Scale has 23-items that encompass 4 sub-scales: 1) physical health (8 items), 2) emotional functioning (5 items), 3) social functioning (5 items), and 4) school functioning (5 items). Items are reverse scored and linearly transformed to a 0 –100 scale, with higher scores indicating better HRQL. Scales are comprised of parallel child self-report and parent proxy-report formats. The parent proxy-report forms are designed to assess the parents’ perceptions of their child’s HRQL. Healthy norms have been established in a cohort of 400 children and 717 parents in the United States [9].

The PedsQL™ Multidimensional Fatigue Scale is an 18-item self-report scale that includes three subscales: general (6 items), sleep/rest fatigue (6 items), and cognitive fatigue (6 items). Similar to the PedsQL™ Multidimensional Generic Core Scale, items on the Fatigue Scale are reverse scored and linearly transformed to a 0 –100 scale, with higher scores indicating less fatigue. Scales are comprised of parallel child self-report and parent proxy-report formats. Healthy U.S. norms have been established in a cohort of 42 children and 102 parents [7].

Overall control of CAH was measured with bone age Z-scores. In addition to monitoring growth and 17-hydroxyprogesterone and androstenedione levels at every visit, bone age films were obtained every 6 months. Change in bone age in relation to chronological age is a valid predictor of overall adrenal androgen control in response to treatment with hydrocortisone [10]. For this study, the most recent bone age films were obtained within 1 year of the questionnaires and were read using the Greulich and Pyle method. The bone age Z-scores were then calculated using the bone age and the standard deviation for each child’s sex and chronological age. The same was done for height Z-scores.

### Statistical analysis

SPSS Version 22 was used to perform the statistical analysis. The mean scores for the general pediatric population for each functional domain on the PedsQL™ Generic Core Scale [9] and the PedsQL™ Fatigue Scale questionnaires [7] were compared to the mean scores of children with CAH. A two-sample t-test was used to compare mean scores between children with CAH and published healthy norms and between parents of children with CAH and parents of published healthy norms [7, 9]. Differences in PedsQL™ (Generic and Fatigue) scores between children with CAH and their parents were also evaluated using a t-test. A one-way between groups analysis of variance was used to determine if PedsQL™ (Generic and Fatigue) scores differed by gender and CAH subtype. Relationships between child and parent ratings of HRQL or Fatigue QoL and hydrocortisone doses, both morning dose (mg/m^2^/day) and total daily dose (mg/m^2^/day), were examined using Pearson’s Correlation Coefficient. The correlations between bone age Z-scores and PedsQL™ (Generic and Fatigue) scores, and between height Z-scores and PedsQL™ (Generic and Fatigue) scores, were also measured using Pearson’s Correlation Coefficient. A *p*-value of 0.05 was used as the significance level for each comparison and each correlation.

## Results

Children with CAH did not report lower scores on the overall PedsQL™ Generic Core or subscales compared to healthy child norms [Table 2] and even rated their physical health higher than healthy children (95% CI (2.10, 10.14), *p* = 0.0.

**Table 2:** Peds QoL Generic and Fatigue Scores: In relation to norms

QoL Score, Mean	Children with CAH	Healthy Children	Mean difference (95% CI)	Parents, CAH Children	Parents, Healthy Children	Mean difference (95% CI)
Generic QoL Scores *(Overall and subscales)*						
Overall	82.71 (*n* = 32)	83.00 (*n* = 401)	−0.29 (−.25, 5.67)	83.90 (*n* = 43)	87.61 (*n* = 717)	−3.71 (−7.37, −0.05)^a^
Physical	90.53 (*n* = 32)	84.41 (*n* = 400)	6.12 (2.10, 10.14)^a^	89.10 (*n* = 44)	89.32 (*n* = 717)	−0.22 (−3.52, 3.08)
Emotional	80.16 (*n* = 32)	80.86 (n = 400)	−0.70 (−6.96, 5.55)	75.90 (*n* = 44)	82.64 (*n* = 717)	−6.74 (−12.36, −1.12)^a^
Social	85.16 (*n* = 32)	87.42 (*n* = 399)	−2.26 (−10.11, 5.58)	90.60 (*n* = 43)	91.56 (*n* = 716)	−0.96 (−4.57, 2.65)
School	75.00 (*n* = 32)	78.63 (*n* = 386)	−3.63 (−9.78, 2.52)	78.99 (*n* = 40)	85.47 (*n* = 611)	−6.48 (−12.75, −0.21)^a^
Fatigue Scores *(Overall and subscales)*						
Overall	79.08 (*n* = 32)	80.49 (*n* = 52)	−1.41 (−7.85, 5.02)	81.90 (*n* = 42)	89.63 (*n* = 102)	−7.73 (−12.24, −3.22)^a^
General	82.81 (*n* = 32)	85.34 (*n* = 52)	−2.53 (−9.13, 4.07)	82.40 (*n* = 42)	89.30 (*n* = 102)	−6.90 (−12.34, −1.46)^a^
Sleep/Rest	74.48 (*n* = 32)	75.00 (*n* = 52)	−0.52 (−8.30, 7.25)	81.60 (*n* = 42)	88.86 (*n* = 102)	−7.26 (−12.14, −2.38)^a^
Cognitive	79.97 (*n* = 31)	81.14 (*n* = 52)	−1.17 (−10.71, 8.37)	81.60 (*n* = 42)	90.72 (*n* = 102)	−9.12 (−15.72, −2.52)^a^

^a^Indicates a significant finding at the 0.05 significance level

*CAH* congenital adrenal hyperplasia, *QoL* quality of life

However, parents’ reports of their children with CAH were significantly lower on the overall PedsQL™ Generic Core (95% CI (−7.37, −0.05), *p* = 0.05), and on the emotional functioning (95% CI (−12.36, −1.12), *p* = 0.02) and the school functioning subscales (95% CI (−12.75, −0.21), *p* = 0.04) than parents’ reports of healthy norms [Table 2]. Children with CAH and their parents did not differ significantly in how they rated the child’s overall QoL on the PedsQL™ Generic Core and subscales [Table 3].

**Table 3:** Peds QoL Generic and Fatigue Scores: Comparing the children with CAH to their parents

QoL Score, Mean	Children with CAH	Parents of CAH Children	Mean difference (95% CI)
Generic QoL Scores *(Overall and subscales)*			
Overall	82.91 (*n* = 30)	83.30 (*n* = 30)	−0.39 (−5.81, 5.03)
Physical	90.42 (*n* = 31)	89.11 (*n* = 31)	1.31 (−4.68, 7.30)
Emotional	80.32 (*n* = 31)	75.97 (*n* = 31)	4.35 (−1.36, 10.07)
Social	85.50 (*n* = 30)	90.33 (*n* = 30)	−4.83 (−13.53, 3.86)
School	74.67 (*n* = 30)	77.33 (*n* = 30)	−2.67 (−9.23, 3.91)
Fatigue Scores *(Overall and subscales)*			
Overall	79.77 (*n* = 30)	82.36 (*n* = 30)	−2.59 (−6.99, 1.81)
General	83.33 (*n* = 31)	82.12 (*n* = 31)	1.21 (−3.50, 5.92)
Sleep/Rest	75.80 (*n* = 31)	82.52 (*n* = 31)	−6.72 (−11.92, −1.52)^a^
Cognitive	80.14 (*n* = 30)	82.36 (*n* = 30)	−2.22 (−9.64, 5.20)

^a^Indicates a significant finding at the 0.05 significance level

*CAH* congenital adrenal hyperplasia, *QoL* quality of life

Children with CAH did not report lower scores on the overall PedsQL™ Fatigue total or subscales compared to healthy child norms [Table 2]. However, parents of children with CAH reported significantly lower scores on the overall PedsQL™ Fatigue total (95% CI (−12.24, −3.22), *p* = 0.001) and the general fatigue (95% CI (−12.34, −1.46), *p* = 0.01), sleep/rest fatigue (95% CI (−12.14, −2.38), *p* = 0.005), and cognitive fatigue subscales (95% CI (−15.72, −2.52), *p* = 0.008) than parents of healthy norms [Table 2]. Children with CAH and their parents did not differ significantly in how they rated the child’s overall QoL on the PedsQL™ Fatigue total and subscales except on the sleep/rest fatigue subscale, where children reported poorer sleep/rest fatigue than their parents (95% CI (−11.92, −1.52), *p* = 0.01) [Table 3].

**Fig. 1:**
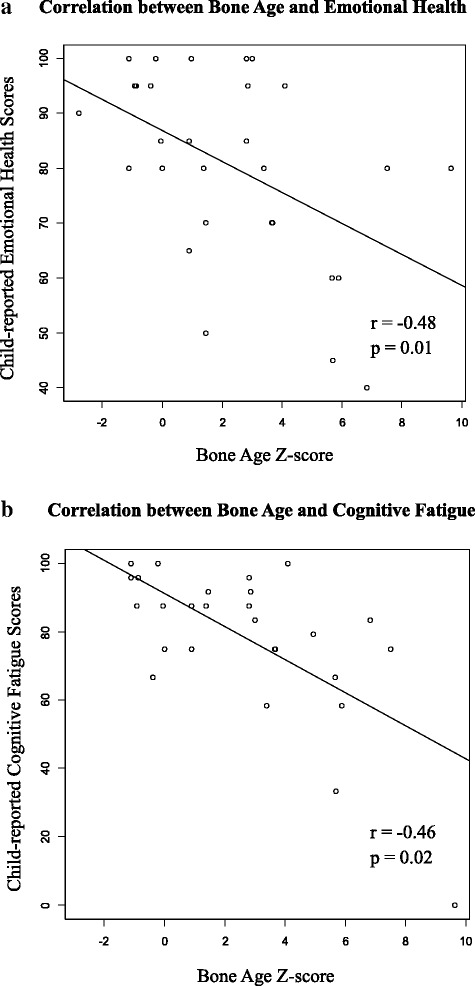
**a** displays the correlation between bone age Z-scores and child-reported emotional health, and **b** displays the correlation between bone age Z-scores and child-reported cognitive fatigue scores in children with CAH

PedsQL™ Generic and Fatigue scores did not differ by sex, CAH subtype, morning or total hydrocortisone doses (mg/m^2^/day), or height Z-scores. Child and parent PedsQL™ Generic and Fatigue scores were examined for differences by sex, CAH subtype, morning or total hydrocortisone doses (mg/m^2^/day), and height Z-scores; no significant differences were found. However, among children with CAH, there was a negative correlation between bone age Z-scores and child-reported scores on the following two subscales: emotional health (*p* = 0.01) from the PedsQL™ Generic Core and cognitive fatigue (*p* = 0.02) from the PedsQL™ Fatigue Scale [Fig. 1].

## Discussion

As healthcare shifts to a more patient-centered approach, HRQL and measures of fatigue become more important measures of health outcomes [11, 12]. HRQL questionnaires are short, validated screening tools that are particularly useful when treating children with chronic conditions, such as CAH, to understand how the disease and therapy impact physical, emotional, and social functioning [13]. There are only two previous studies that examined QoL in children with CAH using validated HRQL screening tools [3, 4]. However, these studies did not examine the relationship between QoL scores and hydrocortisone dosing or chronic androgen exposure, as measured by bone age Z-scores. There are no studies in children with CAH that have examined fatigue using a standardized multidimensional assessment.

In contrast to the two previous reports [3, 4], our pediatric participants with CAH did not report lower overall PedsQL™ Generic scores than healthy norms. In fact, they scored significantly higher on the physical health subscale. What may account for the differences with other studies is that our children with CAH were only on hydrocortisone, which has been found to have less of a negative impact on QoL in adults than other glucocorticoids [14]. In addition, the children with CAH in our study received circadian hydrocortisone administration, with the highest dose given in the early morning hours. Circadian hydrocortisone delivery has been shown to improve QoL in adults with adrenal insufficiency and CAH [15, 16].

Similar to findings from the other two studies [3, 4], parents of children with CAH in our study reported a negative impact that the disease had on their children. Parents also reported significantly lower overall QoL, emotional health, and school functioning scores on the PedsQL™ Generic Core Scale compared to the parents of healthy norms.

Gilban, et al., a cross-sectional study of 25 participants with CAH (mean age 11.4 years), found that children with CAH reported lower overall QoL and lower physical and psychosocial QoL (encompassing social, emotional, and academic scores) [3]; however, only three of the participants were treated with hydrocortisone. The rest with prednisone or dexamethasone. This study found that bone age was on average 2.8 (2.2) years advanced when compared with chronological age but did not specifically examine how QoL varies by advanced bone age or bone age Z-scores, as our study did. Similar to our study, Gilban, et al., did not find any differences in QoL scores between sex and CAH subtypes, and parents of children with CAH reported lower physical and psychosocial scores for their children than parents of healthy norms.

Yau, et al., examined QoL in 33 children with CAH and compared these results to healthy norms and 14 children with hypothyroidism [4]. Children with CAH reported lower scores in overall QoL and in all of the subcategories: physical, emotional, social, and academic when compared with healthy norms. Children with CAH also reported lower school performance than children with hypothyroidism which was thought to be due to the number of missed days of school for illness and doctor appointments. Parents of children with CAH also reported lower scores for overall QoL and emotional functioning for their children than parents of healthy norms, but there was no significant difference found between scores of parents of children with CAH and parents of children with hypothyroidism. This study did not report which glucocorticoids the children with CAH were taking.

In our study, we also found no difference in PedsQL™ Fatigue scores between children with CAH and healthy norms and no difference between children with CAH and their parents except in the sleep/rest PedsQL™ Fatigue subscale, where children reported significantly lower scores. A possible explanation for similar fatigue scores between children with CAH and healthy norms could be that the baseline expectation of “feeling normal” may be lower in children with CAH, as it reflects the way they have felt since birth. Children with late onset acquired adrenal insufficiency, for example, can compare a perceived change in QoL before and after disease onset, whereas children with CAH have no other point of reference [17]. This adaptation by children with CAH to their impairment is a phenomenon known to occur in other chronic congenital diseases [18] and has been noted in adults with CAH, who reported less fatigue compared to adults with other forms of adrenal insufficiency diagnosed later in life [19]. It is also possible the higher adrenal androgen levels in children with CAH compared to controls may ameliorate the fatigue associated with hypocortisolemia. The lower sleep/rest fatigue subscale score reported by children with CAH when compared to their parents may be attributed to the evening hydrocortisone dose. Based on diurnal cortisol secretion, levels between 6 pm and 12 am are at their nadir in healthy children [20], which is during the time frame that children with CAH are typically getting their evening dose (8-10 pm). This results in a cortisol peak during a time that cortisol should be at its lowest based on the physiologic cortisol circadian rhythm. Children with higher cortisol levels before bedtime have been shown to have shorter duration of sleep and lower sleep efficiency [21, 22].

Parents of children with CAH reported significantly lower total and all subscale scores on the PedsQL™ Fatigue Scale than parents of healthy norms. This is not surprising as parents of children with chronic diseases, in general, have been found to over-report poorer QoL in their children, and they may be reporting their own feelings of having a child with a long term medical condition requiring therapy [12].

One Dutch study using non-validated, self-designed QoL questionnaires showed that parents of children with CAH tended to report sleepiness and decreased energy in their children, similar to findings in our study [23]. The majority of children in this study were taking hydrocortisone.

While we found no correlation between daily hydrocortisone dose (morning or total), sex, disease subtype, or height Z-scores and PedsQL™ Generic and Fatigue scores, we did find that higher bone age Z-scores, indicating more advanced bone age and increased androgen exposure, correlated with lower scores on the emotional health and cognitive fatigue subscales among children with CAH. Although one would expect that lower hydrocortisone doses would be associated with increased androgen exposure and lower scores, the lack of correlation between hydrocortisone dose and PedsQL™ Generic and Fatigue scores suggest that inter-individual glucocorticoid sensitivity and cortisol kinetics should be considered when determining the best treatment regimen for these patients.

One limitation of this study is our use of standardized norms. Although standardized, environmental factors may play a role in QoL, so the results may not exactly reflect those of healthy controls living in Minnesota.

## Conclusion

Our study found that although children may not see the effect that their chronic disease has on their lives, their parents do see the impact of CAH. PedsQL™ Generic and Fatigue scores did not differ by sex, CAH subtype, morning or total hydrocortisone doses (mg/m2/day), or height Z-scores. However, negative associations between bone age Z-scores and emotional health or cognitive fatigue suggest an impact from chronic hypocortisolemia and hyperandrogenemia. More studies to specifically look at circadian versus non-circadian administration of hydrocortisone, and androgen and hydrocortisone exposure over the course of the day through pharmacokinetics and pharmacodynamics studies and its effects on HRQL are needed.

## Abbreviations

CAH: Congenital adrenal hyperplasia
HRQL: Health-related quality of life
QoL: Quality of life
